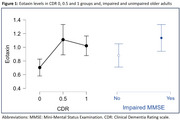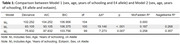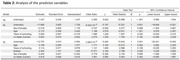# Relationship between cerebrospinal fluid eotaxin levels and cognitive decline: results of a Brazilian Cohort Study

**DOI:** 10.1002/alz70855_103548

**Published:** 2025-12-23

**Authors:** Joice Coutinho de Alvarenga, Ivonne Carolina Bolaños Burgos, Gabriela Tomé Oliveira Engelmann, Júlia de Almeida, Giovanna Correia Pereira Moro, Erika de Oliveira, Natália Silva Dias, Debora Marques de Miranda, Marco Aurélio Romano‐Silva, Jonas Jardim de Paula, Bernardo de Mattos Viana, Maria Aparecida Camargos Bicalho

**Affiliations:** ^1^ Universidade Federal de Minas Gerais, Belo Horizonte, Brazil; ^2^ Sciences Applied to Adult Health Postgraduate Program, School of Medicine, Universidade Federal de Minas Gerais (UFMG), Belo Horizonte, Minas Gerais, Brazil; ^3^ Cog‐Aging Research Group, Universidade Federal de Minas Gerais (UFMG), Belo Horizonte, Minas Gerais, Brazil; ^4^ Older Adult's Psychiatry and Psychology Extension Program (PROEPSI), School of Medicine, Universidade Federal de Minas Gerais (UFMG), Belo Horizonte, Minas Gerais, Brazil; ^5^ Cog‐Aging Research Group, Belo Horizonte, Minas Gerais, Brazil; ^6^ Molecular Medicine Program, School of Medicine, Federal University of Minas Gerais, Belo Horizonte, Minas Gerais, Brazil; ^7^ Molecular Medicine Postgraduate Program, School of Medicine, Universidade Federal de Minas Gerais (UFMG), Belo Horizonte, Minas Gerais, Brazil; ^8^ Undergraduate Medicine, Faculty of Medicine, Universidade Federal de Minas Gerais (UFMG), Belo Horizonte, Minas Gerais, Brazil; ^9^ Universidade Federal de Minas Gerais, Belo Horizonte, Minas Gerais, Brazil; ^10^ Jenny de Andrade Faria Institute – Outpatient Reference Center for the Elderly, Universidade Federal de Minas Gerais (UFMG), Belo Horizonte, Minas Gerais, Brazil; ^11^ Geriatrics and Gerontology Center Clinical Hospital of Universidade Federal de Minas Gerais, Belo Horizonte, Minas Gerais, Brazil; ^12^ Older Adult Psychiatry and Psychology Extension Program (PROEPSI), Faculty of Medicine, Universidade Federal de Minas Gerais (UFMG), Belo Horizonte, Minas Gerais, Brazil; ^13^ Department of Mental Health, Faculty of Medicine, Universidade Federal de Minas Gerais (UFMG), Belo Horizonte, Minas Gerais, Brazil; ^14^ Department of Psychiatry, School of Medicine, Federal University of Minas Gerais, Belo Horizonte, Minas Gerais, Brazil; ^15^ Neurotec R National Institute of Science and Technology (INCT‐Neurotec R), Faculty of Medicine, Universidade Federal de Minas Gerais (UFMG), Belo Horizonte, Minas Gerais, Brazil; ^16^ Older Adult's Psychiatry and Psychology Extension Program I Federal University of Minas Gerais, Belo Horizonte, MG, Brazil; ^17^ Older Adult's Psychiatry and Psychology Extension Program Federal University of Minas Gerais, Belo Horizonte, Minas Gerais, Brazil; ^18^ Geriatrics and Gerontology Center Clinical Hospital of University of Minas Gerais, Belo Horizonte, Minas Gerais, Brazil; ^19^ Hospital das Clínicas da UFMG, University Hospital, Universidade Federal de Minas Gerais (UFMG), Belo Horizonte, Minas Gerais, Brazil; ^20^ Jenny de Andrade Faria Institute – Outpatient Reference Center for the Elderly, Universidade Federal de Minas Gerais (UFMG), Belo Horizonte, Minas Gerais, Brazil; ^21^ Federal University of Minas Gerais, Belo Horizonte, Minas Gerais, Brazil; ^22^ Department of Internal Medicine, School of Medicine, Federal University of Minas gerais, Belo Horizonte, Minas Gerais, Brazil; ^23^ Department of Clinical Medicine, Faculty of Medicine, Universidade Federal de Minas Gerais (UFMG), Belo Horizonte, Minas Gerais, Brazil

## Abstract

**Background:**

Previous evidence suggests that eotaxin‐1 could suppress neurogenesis in the hippocampus in mice and has been associated with left temporal medial reduction in humans with mild cognitive impairment (MCI) and dementia.

**Objectives:**

To evaluate the relationship between eotaxin‐1 (CCL11) and cognitive decline in a sample of Brazilian older adults

**Method:**

This is a cross‐sectional study that included 108 older adults participants from the Cog‐Aging Cohort Study, evaluated from 2023 to 2024. All participants underwent cognition assessment and cerebrospinal fluid collection (CSF). Cytokines were measured using the Luminex xMAP technique. The participants were classified into three groups: cognitive unimpaired (CU), MCI, and dementia, according to CDR score, and clinical evaluation. Mini‐Mental State Examination (MMSE) cut‐off scores were 19/20 for illiterate and 23/24 for literate participants. We used the Spearman's test; Mann‐Whitney test; Kruskal‐Wallis with Dunn's Post‐Hoc analyses; and the logistic regression (LR) with age, years of schooling, sex, APOE carrier status and eotaxin levels. All tests used a significance level of 5%.

**Result:**

The mean age was 74.85 years (SD±6.77); median 4 years (IQR7) of formal education, 63.8% were female and 39.04% were APOEε4 carriers. The MMSE median score was 23 points (IQR6); 19.4% of the participants were in the CU group, 30.6% had MCI and 50% had dementia. There was significant direct correlation between eotaxin with CDR (rho=0.195, *p* = 0.04) and inverse correlation with MMSE score (rho=‐0.267, *p* = 0.005). The post‐hoc analyses showed a difference of eotaxin levels between CU group and MCI (*p* = 0.004), and between CU and dementia group (*p* = 0.006). There was also a difference between eotaxin levels between impaired and unimpaired MMSE groups (*p* = 0.027). The LR model with age, years of schooling, sex and APOE could predict 68.57% CU participants from cognitively impaired ones. The inclusion of eotaxin levels to the model improved it, raising the performance to 86.6% with an AUC of 0.834. In the final model, only age (OR= 1.17, *p* = 0.002) and eotaxin levels (OR= 11.07, *p* = 0.029) were significant.

**Conclusion:**

CSF eotaxin levels were lower in cognitively unimpaired participants, as assessed by both CDR and MMSE. These results point to the association of eotaxin and cognitive impairment in MCI and Dementia.